# Traditional Chinese Medicine, Qingfei Paidu Decoction and Xuanfei Baidu Decoction, Inhibited Cytokine Production *via* NF-κB Signaling Pathway in Macrophages: Implications for Coronavirus Disease 2019 (COVID-19) Therapy

**DOI:** 10.3389/fphar.2021.722126

**Published:** 2021-10-26

**Authors:** Yujia Li, Bin Li, Pan Wang, Qinghua Wang

**Affiliations:** ^1^ Institute of Blood Transfusion, Chinese Academy of Medical Sciences and Peking Union Medical College, Chengdu, China; ^2^ The Joint Laboratory on Transfusion-transmitted Diseases (TTD) Between Institute of Blood Transfusion, Chinese Academy of Medical Sciences and Nanning Blood Center, Nanning, China; ^3^ The Traditional Chinese Medicine Hospital of Wenjiang District, Chengdu, China

**Keywords:** COVID-19, traditional Chinese medicine, macrophage, NF-κB signaling pathway, cytokine storm

## Abstract

**Background and Aims:** Qingfei Paidu decoction (QPD) and Xuanfei Baidu decoction (XBD) are two typical traditional Chinese medicines with proven efficacy for the treatment of SARS-CoV-2, although the underlying mechanism is not well defined. Blunted immune response and enhanced production of pro-inflammatory cytokines (cytokine storm) are two main features observed in patients infected with SARS-CoV-2. Analysis based on network pharmacology has revealed that both QPD and XBD played an important role in the regulation of host immunity. We therefore investigated the role of QPD and XBD in the modulation of innate immunity *in vitro*, focusing on the type 1 interferon (IFN) signaling pathway in A549 cells and pro-inflammatory cytokine production in macrophages. **Methods:** A549 cells were treated with QPD or XBD and the production of endogenous IFNα and IFNβ as well as the expression levels of some interferon-stimulated genes (ISGs) were detected by reverse transcriptase-quantitative PCR (RT-qPCR). Macrophages derived from THP-1 cells were treated with QPD or XBD and their pro-inflammatory cytokine expression levels were measured by RT-qPCR, 6 h post LPS stimulation. In addition, the expression levels of some pro-inflammatory cytokines were further analyzed by ELISA. The effect of QPD and XBD on the NF-κB signaling pathway and the pinocytosis activity of THP-1-derived macrophages were evaluated by Western blot and neutral red uptake assay, respectively. **Results:** Although QPD and XBD showed very little effect on the type 1 IFN signaling pathway in A549 cells, either QPD or XBD markedly inhibited the production of pro-inflammatory markers including interleukin-6, tumor necrosis factor-α, monocyte chemotactic protein-1, and chemokine ligand 10 in THP-1-derived M1 macrophages. In addition, the phosphorylation of IκBα and NF-κB p65 during the process of macrophage polarization was significantly suppressed following QPD or XBD treatment. QPD and XBD also suppressed the pinocytosis activity of macrophages. **Conclusion:** QPD and XBD have been shown to have robust anti-inflammatory activities *in vitro*. Our study demonstrated that both QPD and XBD decreased pro-inflammatory cytokine expression, inhibited the activation of the NF-κB signaling pathway, and blunted pinocytosis activity in THP-1-derived macrophages.

## Introduction

Globally, there are more than 154 million confirmed cases of coronavirus disease 2019 (COVID-19), including 3.2 million deaths as of May 6, 2021 (Available online: https://covid19.who.int/). The COVID-19 pandemic is caused by infection with a non-enveloped RNA beta coronavirus, specifically the severe acute respiratory syndrome coronavirus 2 (SARS-CoV-2). SARS-CoV-2 infection, like most other virus infections, triggers the host’s innate immune response which constitutes the first line of defense against invading pathogens. The dysregulation of the innate immune response is closely associated with morbidity and mortality of COVID-19 patients. For example, although it is part of the first line of defense against virus infections, production of type 1 interferon (IFN), one of the key antiviral mediators, is blunted in patients infected with SARS-CoV, which is in contrast to the fact that high levels of type 1 IFN have been detected in patients infected with SARS-CoV (Acharya et al., 2020). Moreover, the potential use of IFNs in COVID-19 therapy (Park et al., 2020) also highlights that impaired systemic IFN production is a crucial determinant in the pathogenesis of SARS-CoV-2 infection. Another characteristic of severe COVID-19 patients is the cytokine storm: over-production of numerous cytokines and chemokines such as interleukin-6 (IL-6), tumor necrosis factor-α (TNF-α), monocyte chemotactic protein-1 (CCL2), and chemokine (C-X-C motif) ligand 10 (CXCL10) (Merad et al., 2020). As one of the most enriched immune cell types in the lungs of COVID-19 patients, macrophages have been shown to contribute to hyper-inflammation that leads to cytokine storms in patients with severe COVID-19. However, the exact contribution of macrophages in the pathogenesis of SARS-Cov-2 remains to be elucidated (Wang C. et al., 2020; Booz et al., 2020; Merad et al., 2020).

Various traditional Chinese medicines (TCM) have been used to treat patients infected with SARS-CoV-2 in China, mainly including oral medication, such as Lianhua Qingwen capsules, Jinhua Qinggan granules, different kinds of decoctions, and TCM injections such as Xuebijing injections and Shenfu injections [reviewed in (Al-Romaima et al., 2020; Luo et al., 2020; Wang et al., 2021b; Luo et al., 2021)]. Among them, two decoctions, the Qingfei Paidu decoction (QPD) and the Xuanfei Baidu decoction (XBD), have been shown to have significant efficacy against SARS-CoV-2 infection (Shi et al., 2020; Xiong et al., 2020; Huang et al., 2021). With an effective rate of over 90% (Al-Romaima et al., 2020), QPD was officially recommended for the treatment of mild, medium, severe, and critical COVID-19 patients in the 7th version of the diagnosis and treatment guidelines issued by the National Health Commission (NHC) of China (Available online: http://www.nhc.gov.cn/xcs/zhengcwj/202003/46c9294a7dfe4cef80dc7f5912eb1989.shtml). XBD granules are also recommended for the treatment of moderate patients (Huang et al., 2021). Network pharmacology analysis revealed that both QPD (Niu et al., 2021) and XBD (Wang Y. et al., 2020) play an important role in regulating host immunity to prevent hyperinflammation, which may result in cytokine storm. Clinical data (Xiong et al., 2020) also showed that C-reactive protein, a non-specific marker of inflammation, was significantly decreased in the XBD-treated group compared to that in the control group. However, little is known about the underlying molecular mechanisms.

Therefore, in the present study, we aimed to investigate the effect of QPD and XBD on the host’s innate immunity, focusing on the type 1 IFN signaling pathway and inflammatory pathway in macrophages.

## Material and Methods

### Cells

Human adenocarcinomic alveolar basal epithelial cell line (A549) and human myeloid leukemia mononuclear (THP-1) cells were purchased from the West China Medical Center of Sichuan University and routinely preserved in our laboratory. The A549 cells were maintained in Dulbecco’s Modified Eagle Medium (DMEM) (Hyclone, United States) supplemented with 10% fetal bovine serum (Gibco, United States), 100 IU/ml ampicillin, and 100 mg/ml streptomycin (Gibco, United States) at 37°C in a 5% CO2 humidified incubator. The THP-1 cells were maintained in RPMI-1640 (Hyclone, United States) medium supplemented with 10% fetal bovine serum (Gibco, United States), 10 mmol/L HEPES (Cellgro, United States), 100 IU/ml ampicillin, and 100 mg/ml streptomycin (Gibco, United States) at 37°C in a 5% CO2 humidified incubator. The THP-1 cells were differentiated into M0 macrophages by 100 ng/ml phorbol-12-myristate-13-acetate (PMA) (Sigma, United States) stimulation for 48h, followed by 24 h rest in RPMI-1640 medium without PMA. The M0 macrophages were primed with fresh culture medium with 20 ng/ml IFN-γ (Peprotech, United States) and 1 µg/ml Escherichia coli 0111:B4 lipopolysaccharide (LPS) (Sigma, United States) for M1 polarization as previously reported by Chanput et al. (2014).

### Decoction Preparation

The preparation processes for QPD and XBD are exactly the same. The drugs (raw materials) were soaked in 500 ml of pure water for 30 min and then boiled until 300 ml of liquid remained, which was collected by filtration as the first part. Another 300 ml of pure water was added, to the dregs and then the mixture was boiled slowly until 200 ml of liquid remained, which was collected by filtration as the second part and mixed well with the first part to obtain an approximately 500 ml decoction. The decoction was centrifuged at 5,000 rpm (4,109 × g) for 30 min at room temperature and the supernatant was collected and filtered by 0.22 µm polypropylene microporous membrane and stored at −80°C until use. The QPD was concentrated to a density of 1.02 g/ml and the XBD was concentrated to 0.98 g/ml. The raw materials of QPD and XBD are listed in Table 1 and Table 2, respectively. Previous studies reported that 129 compounds have been identified in QPD by liquid chromatography quadrupole-time of flight mass spectrometry analysis (Yang et al., 2020; Wang et al., 2021b). Among them, eight specific compounds were identified as potential candidates which may directly interact with the SARS-CoV-2 viral proteins.

**TABLE 1:** Raw materials of Qingfei Paidu decoction.

Name	Chinese name	Medicinal parts	Quantities (g)
*Ephedra sinica* Stapf	Ma Huang	Stem	9
*Glycyrrhiza uralensis* Fisch.ex DC.	Zhi Gan Cao	Root	6
*Prunus armeniaca* L	Xing Ren	Seed	9
*Gypsum fibrosum*	Sheng Shi Gao	*	30
*Atractylodes macrocephala* Koidz	Bai Zhu	Root	9
*Bupleurum chinense* DC.	Chai Hu	Root	16
*Scutellaria baicalensis* Georgi	Huang Qin	Root	6
*Pinellia ternate* (Thunb.) Makino	Jiang Ban Xia	Root	9
*Aster tataricus* L.F.	Zi Wan	Root	9
*Tussilago farfara* L.	Kuan Dong Hua	Flower	9
*ris domestica* (L.) Goldblatt and Mabb	She Gan	Root	9
*Asarumsieboldii* Miq	Xi Xin	Whole plant	6
*Dioscorea opposite* Thunb	Shan Yao	Root	12
*Citrus × aurantium* L	Zhi Shi	Fruit	6
*Pogostemon cablin* (Blanco) Benth	Huo Xiang	Whole plant	9
*Zingiber officinale* Rosc	Sheng Jiang	Root	15
*Poria cocos* (Schw.) Wolf	Fu Ling	whole	15
*Citrus × aurantium* L	Chen Pi	Fruit	6
*Cinnamomum cassia* (L.) J.Pres*l*	Gui Zhi	Stem	9
*Alisma orientalis* (Sam.) Juzep	Ze Xie	Root	9
*Polyporus umbellatus* (Pers.) Fries	Zhu Ling	whole	9

*, Sheng Shi Gao (*Gypsum fibrosum*) is an inorganic substance. All botanical drugs in Qingfei Paidu decoction were fully validated using http://www.plantsoftheworldonline.org/

**TABLE 2:** Raw materials of Xuanfei Baidu decoction

Name	Chinese name	Medicinal parts	Quantities (g)
*Ephedra sinica* Stapf	Ma Huang	Stem	6
*Prunus armeniaca* L	Xing Ren	Seed	15
*Gypsum fibrosum*	Sheng Shi Gao	*	30
*Coix lacryma-jobi* L	Yi Yi Ren	Seed	*30*
*Atractylodes lancea* (Thunb.) DC.	Cang Zhu	Root	10
*Pogostemon cablin* (Blanco) Benth	Huo Xiang	Whole plant	15
*Artemisia annua* L	Qing Hao	Whole plant except root	12
*Citrus × reticulata* Blanco	Ju Hong	Fruit	15
*Glycyrrhiza uralensis* Fisch.ex DC.	Zhi Gan Cao	Root	10
*Phragmites communis* Trin	Lu Gen	Root	30
*Lepidium apetalum* Willd	Ting Li Zi	Seed	15
*Verbena officinalis* L	Ma Bian Cao	Whole plant except root	30
*Reynoutria japonica* Houtt	Hu Zhang	Root	20

*, Sheng Shi Gao (*Gypsum fibrosum*) is an inorganic substance. All botanical drugs in Xuanfei Baidu decoction were fully validated using http://www.plantsoftheworldonline.org/

### Cytotoxicity Assay

The cytotoxic effect of QPD and XBD on A549 and THP-1 cells were evaluated with Cell Counting Kit-8 (CCK-8) (Biosharp, China), following the manufacturer’s instructions. Briefly, monolayers of A549 cells or M0 THP-1 macrophages in 96-well plates were incubated with indicated concentrations of QPD or XBD. The cells were rinsed with phosphate-buffered saline (PBS) (Hyclone, United States) at 0, 24, 48, 72, and 96h, followed by staining with 10ul of CCK8 solution per well. The absorbance was measured at 450 nm using a Multiskan Spectrum reader (Thermo Fisher, United States).

RNA Isolation and Reverse Transcriptase-quantitative PCR Analysis (RT-qPCR).

The total intracellular RNA was extracted using Trizol (Invitrogen, United States) and quantified by NanoDrop (Thermo, United States). Reverse-transcription was carried out using Rever TraAceq PCR RT Master Mix (TOYOBO, Japan) following the manufacturer’s recommended protocol. The resulting cDNA was amplified by NovoStart SYBR qPCR SuperMix Plus (Novoprotein, China). Primers for quantitative PCR are listed in Table 3.

**TABLE 3:** Primers used for real-time PCR.

Gene name	Nucleotide sequence	Gene name	Nucleotide sequence
GAPDH	F: 5′-GCC​TCC​TGC​ACC​ACC​AAC​TG-3′	IFIT-1	F: 5′- GCA​GCC​AAG​TTT​TAC​CGA​AG-3′
	R: 5′-ACG​CCT​GCT​TCA​CCA​CCT​TC-3		R: 5′- GCC​CTA​TCT​GGT​GAT​GCA​GT-3′
ACE2	F: 5′-AAC​TGC​TGC​TCA​GTC​CAC​C-3′	IL-6	F: 5′-ATG​CCT​GAC​CTC​AAC​TCC​ACT-3′
	R: 5′-AAA​AGG​CAG​ACC​ATT​TGT​CCC-3		R: 5′-GCC​ACC​CAG​CTG​CAA​GAT​TTC-3′
TMPRSS2	F: 5′-CCT​GTG​TGC​CAA​GAC​GAC​TG-3′	TNF-α	F: 5′-AGC​TGC​CAG​GCA​GGT​TCT​CTT​CC-3′
	R:5′-TTATAGCCCATGTCCCTGCAG-3′		R: 5′-GGT​TAT​CTC​TCA​GCT​CCA​CGC​CA-3′
IFNα	F: 5′-TCG​CCC​TTT​GCT​TTA​CTG​AT-3′	CCL2	F: 5′-TCT​GTG​CCT​GCT​GCT​CAT​AG-3′
	R: 5′- GGG​TCT​CAG​GGA​GAT​CAC​AG-3′		R: 5′-TGG​AAT​CCT​GAA​CCC​ACT​TC-3′
IFNβ	F: 5′-AAA​CTC​ATA​GCA​GTC​TGC​A-3′	CXCL10	F: 5′-GCC​TTG​GCT​GTG​ATA​TTG​TG-3′
	R: 5′-AGG​AGA​TCT​TCA​GTT​TCG​GAG​G-3′		R: 5′-TAA​GCC​TTG​CTT​GCT​TCG​AT-3′
MxA	F: 5′-GTG​CAT​TGC​AGA​AGG​TCA​GA-3′	NF-kB	F: 5′- ATG​TGG​AGA​TCA​TTG​AGC​AGC-3′
	R: 5′-CTG​GTG​ATA​GGC​CAT​CAG​GT-3′		R: 5′-CCT​GGT​CCT​GTG​TAG​CCA​TT-3′

GAPDH, glyceraldehyde-3-phosphate dehydrogenase; ACE2, angiotensin-converting enzyme 2; TMPRSS2, transmembrane serine protease; IFN, interferon; MxA, myxovirus resistance 1; IFIT-1, Interferon-induced tetrapeptide repeat protein 1; IL-6, Interleukin-6; TNF-α, tumor necrosis factor-α; CCL2, monocyte chemotactic protein-1; CXCL10, chemokine (C-X-C motif) ligand 10; NF-kB, nuclear factor kappa-B.

### Enzyme-Linked Immunosorbent Assay

The THP-1 monocytes were differentiated into M0 macrophages as described above and treated with decoctions at indicated concentrations for 24 h. The cells were then washed with PBS three times and supplied with phenol-red free RPMI-1640 media with 1 µg/ml LPS and 20 ng/ml IFN-γ. Twenty-four hours later, the culture supernatant was collected for ELISA analysis. The levels of IL-6 and NF-κB were detected by manual IL-6 and NF-κB ELISA kits (Elabscience, China) respectively, following the manufacturer’s instructions.

### Western Blot

The M0 THP-1 macrophages were treated with 5% QPD or 5% XBD for 24 h. Then the cells were stimulated with or without 1 µg/ml LPS and 20 ng/ml IFN-γ. The total intracellular protein was collected at 1 h and 2 h post-stimulation. IκBα, phospho-IκBα, NF-κB p65, and phospho-NF-κB p65 protein levels were assessed by western blot using IκBα (L35A5) mouse mAb, phospho-IκBα (Ser32/36)(5A5) mouse mAb, NF-κB p65 (D14E12) XP^®^ rabbit mAb, and phospho-NF-kB p65 (Ser536) (93H1) rabbit mAb (Cell Signaling Technology, United States), respectively. Secondary antibodies were HRP-labeled goat anti-mouse or anti-rabbit IgG (Proteintech, China). The protein bands were visualized using an ECL chemiluminescent detection kit (Millipore, United States) in an ImageQuant LAS 4000mini (GE, United States).

### Pinocytic Activity Assay

The THP-1 monocytes were seeded in a 96-well plate at the density of 4 × 10^5^cells/ml with 200 μL/well, and the cells were differentiated into M0 macrophages and polarized into M1 macrophages as described above. A neutral red uptake assay was employed to evaluate the pinocytosis function of the macrophages as previously described (Jacobo-Salcedo Mdel et al., ).

### Statistical Analyses

The experiments were repeated three times. The significance of the differences between the grops was assessed using ANOVA (data normality and homogeneity of variance) or the Kruskal-Wallis rank test, where appropriate. *p* < 0.05 was considered statistically significant.

## Results

### Qingfei Paidu Decoction and Xuanfei Baidu Decoction Have Little Effect on the Activation of the Type 1 IFN Signaling Pathway in A549 Cells

**FIGURE 1:**
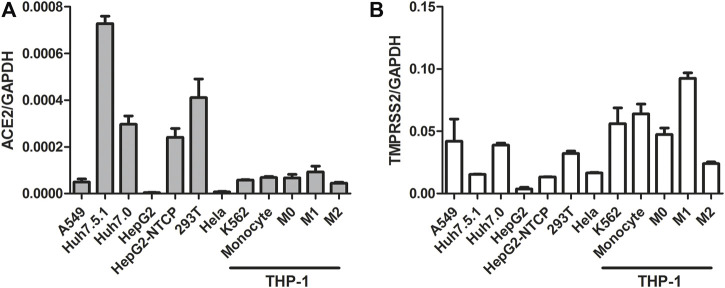
ACE2 and TMPRSS-2 expression in different cell lines. Seed cells to be 80–90% confluent in 6-well plate until cells were harvested and total RNAs were extracted. 1ug total RNA was applied for reverse transcription. ACE2 and TMPRSS-2 expression was assessed using real-time PCR (normalized to GAPDH). Data are presented as mean ± SD (n ≥ 3).

**FIGURE 2:**
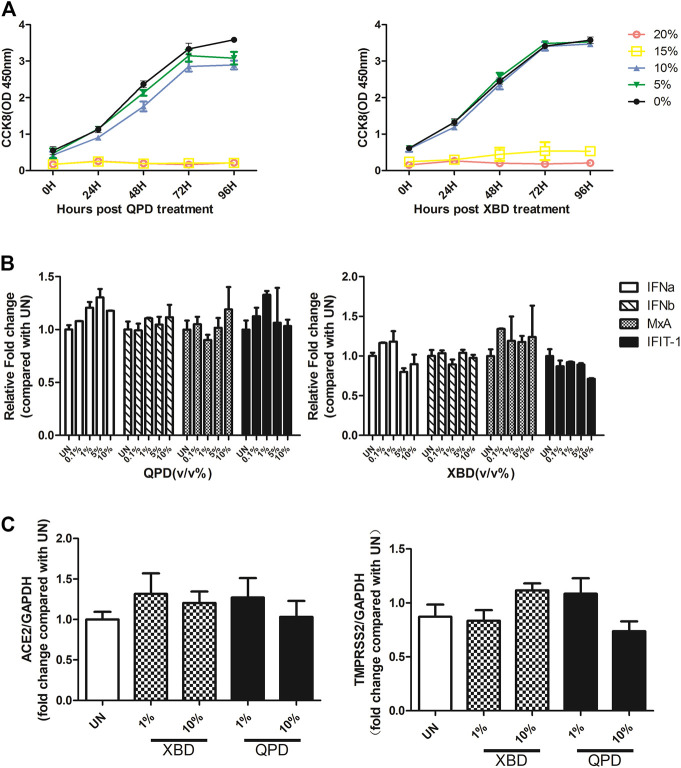
Effect of QPD and XBD on IFN signaling pathway, ACE2, and TMPRSS-2 in A549 cells. **(A):** Cytotoxic effect of QPD (left) and XBD (right) on A549 cells. A549 cells were seeded at 6 × 10^5^/ml, 2 ml per well in 6-well plates for 24 h before QPD or XBD was added into each well at indicated concentrations (%, v/v). 48 h post QPD or XBD treatment, total RNA was extracted to detect IFNα, IFNβ, MxA, IFIT-1 mRNAs **(B)** or ACE2 and TMPRSS-2 mRNAs **(C)** by RT-qPCR. UN, untreated control. Data are presented as mean ± SD (*n* ≥ 3).

Activation of the IFN signaling pathway in host cells is one of the important immune responses to viral infections. The fact that SARS-CoV-2 blunts the host’s innate immune response and is characterized by weak IFN production indicates that SARS-CoV-2 may target the IFN pathway as part of its strategy to avoid being eliminated by innate immunity. Thus, we first investigated the effect of QPD and XBD on type 1 IFN signaling. Cells expressing both angiotensin-converting enzyme 2 (ACE2) and transmembrane serine protease (TMPRSS)-2 are the main targets during SARS-CoV-2 infection. Therefore, the A549 cell, a human lung epithelial cell line with both ACE2 (Figure 1A) and TMPRSS-2 (Figure 1B) expression, was selected as the cell model for this study. Consistent with Qi et al. (2020), the endogenous expression level of ACE2 was very low, although it could be expressed in multiple organs and tissues. The cell viability after QPD or XBD treatment was determined with CCK-8 kits. Both QPD (Figure 2A, left) and XBD (Figure 2A, right) showed no apparent cytotoxicity for A549 cells at concentrations up to 15% (v/v). However, neither QPD (Figure 2B, left) nor XBD (Figure 2B, right) showed any effect on IFNα, IFNβ, or ISG expression (RT-qPCR), indicating that the endogenous production of type 1 IFNs and subsequent (down-stream) ISGs expression were not activated by these two decoctions. Moreover, no significant difference of ACE2 nor TMPRSS-2 expression was found between the decoction-treated group and the control group (Figure 2C).

### Qingfei Paidu Decoction and Xuanfei Baidu Decoction Significantly Inhibit Cytokine Production in THP-1-Derived Macrophages

**FIGURE 3:**
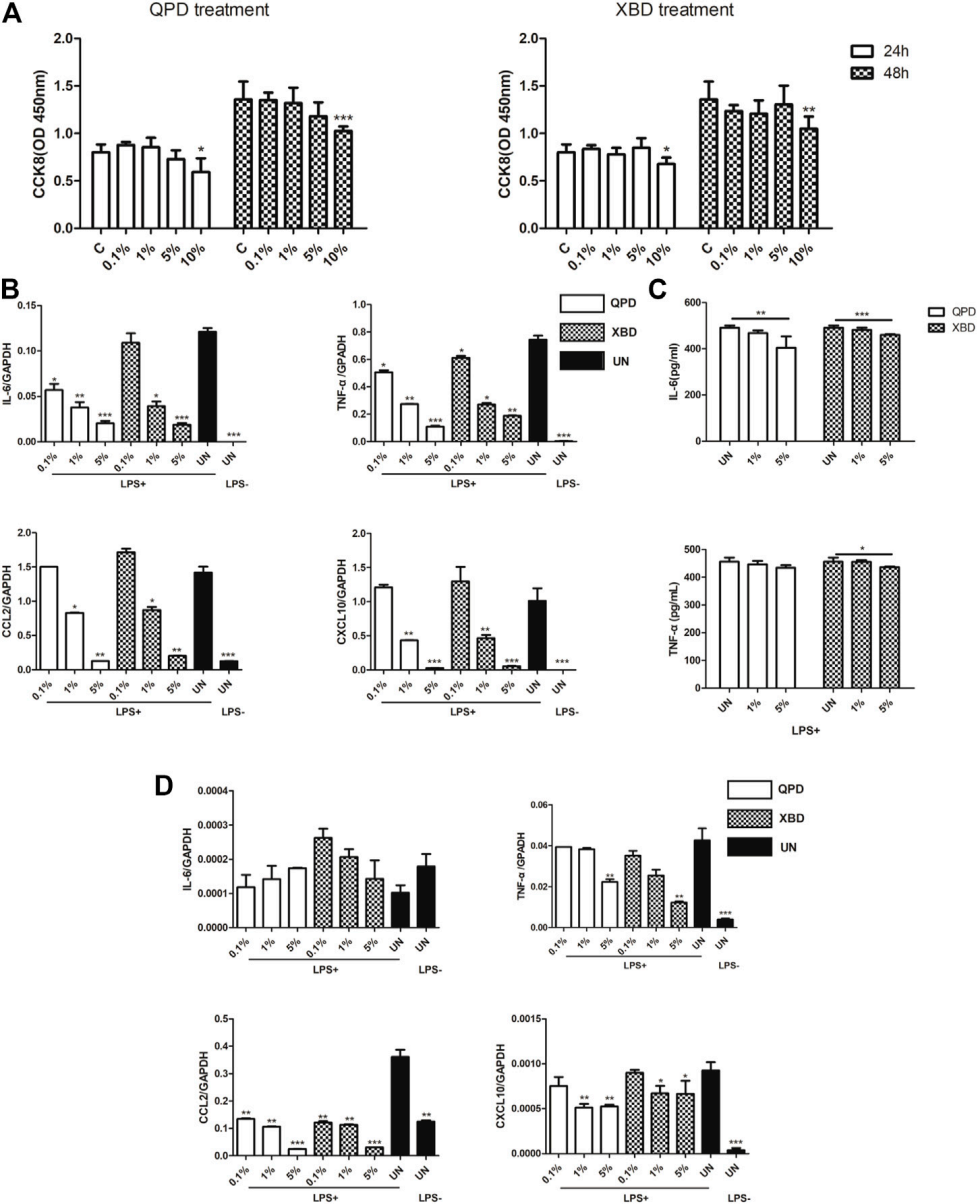
QPD and XBD inhibited inflammatory cytokines expression in LPS-stimulated THP-1 macrophages. Cytotoxic effect of QPD (left) and XBD (right) on THP-1 macrophages **(A)**. THP-1 monocytes were seeded at 0.5 × 10^6^/ml, 2 ml per well in 6-well plates and differentiated into M0 macrophages before QPD or XBD was added into each well at indicated concentrations (%, v/v). 24 h post QPD or XBD treatment, the supernatant was removed and the cells were washed with PBS three times and incubated in the medium supplied with or without 1 µg/ml LPS and 20 ng/ml IFN-γ. 6 h later, the total RNA was extracted to detect IL-6, TNF-α, CCL2, and CXCL10mRNA levels by RT-qPCR **(B)**. 24 h later, the supernatant was collected to analyze IL-6 and TNF-α by ELISA **(C)**. M0 THP-1 macrophages were treated with or without 1 µg/ml LPS and 20 ng/ml IFN-γ for 6 h and then treated with QPD or XBD at indicated concentrations for 24 h. Total RNA was extracted to detect IL-6, TNF-α, CCL2, and CXCL10 mRNA levels by RT-qPCR **(D)**. C and UN, untreated control. LPS+, treated with LPS and IFN-γ; LPS-, treated without LPS and IFN-γ. Data are presented as mean ± SD (*n* ≥ 3). * p<0.005 ; ** p<0.001 ; *** p<0.001 compared with C or UN + LPS group or as indicated.

THP-1 is a cell model widely used to explore macrophage function and inflammatory response pathways. THP-1 cells can be differentiated into mature macrophages with relatively high similarity to human peripheral blood mononuclear cells (PBMCs) and monocyte-derived macrophages (Chanput et al., 2014). It was reported that SARS-CoV-2 could infect peripheral blood monocytes and promote ACE2 expression (Codo et al., 2020). Consistent with that study, THP-1-derived macrophages also showed significant levels of ACE2 and TREMPSS-2 expression in the M0, M1, and M2 stages (Figure 1). The results from CCK-8 tests indicated that both QPD (Figure 3A, left) and XBD (Figure 3A, right) showed no apparent cytotoxicity for THP-1-derived macrophages at concentrations up to 10% (v/v). To determine the effect of QPD and XBD on the expression of cytokines and chemokines in M1-like inflammatory macrophages, we treated the M0 THP-1 macrophages with QPD or XBD for 24 h and analyzed the mRNA expression levels of some typical cytokines and chemokines 6 h after LPS stimulation. The results from RT-qPCR analysis showed that both QPD and XBD significantly inhibited the expression of IL-6, TNF-α, CCL2, and chemokine (C-X-C motif) ligand 10 (CLCX10) (Figure 3B), and this inhibition effect was further confirmed by ELISA (Figure 3C). Similar results were obtained when the M0 THP-1-derived macrophages were stimulated by LPS for 6 h first and then treated with QPD or XBD for 24 h (Figure 3D).

### Qingfei Paidu Decoction and Xuanfei Baidu Decoction Inhibited the Activation of the NF-κB Signaling Pathway and the Pinocytosis Activity of THP-1-Derived Macrophages

**FIGURE 4:**
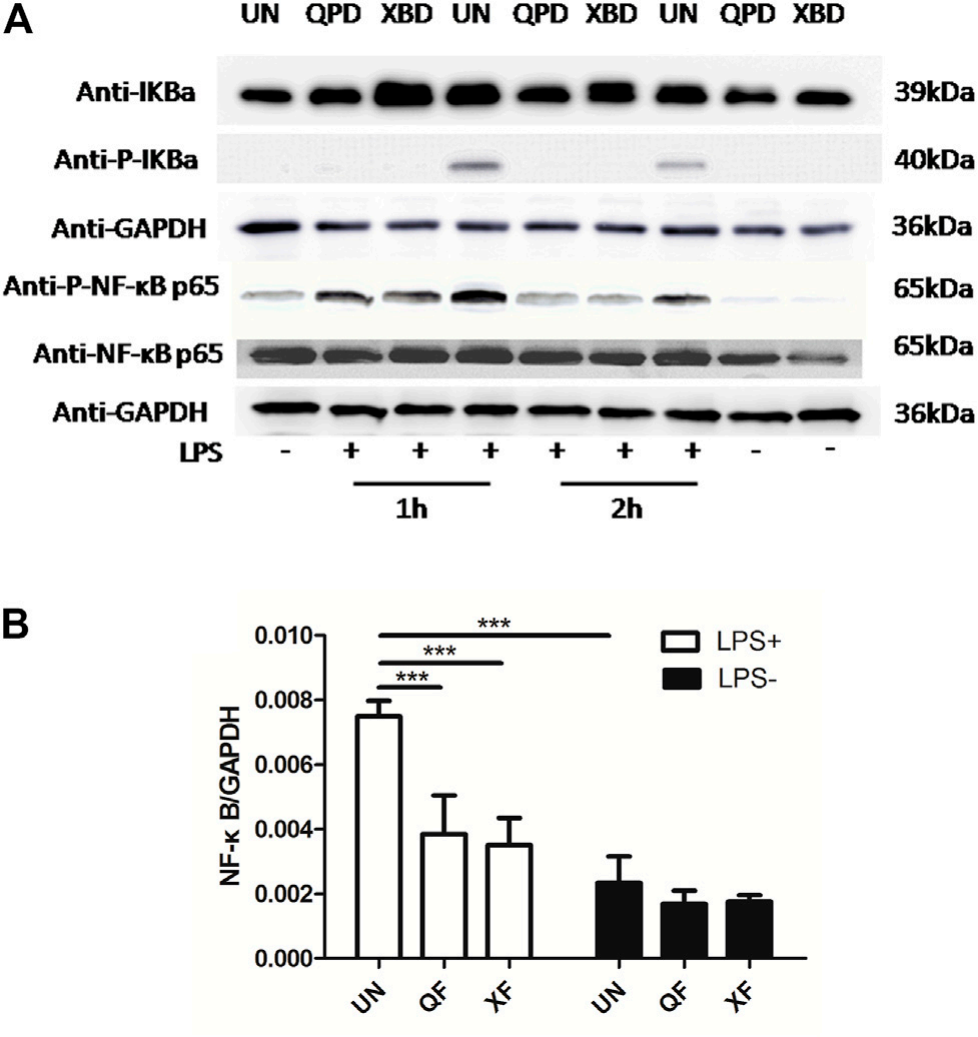
QPD and XBD prevented LPS-induced activation of NF-κB signaling pathway in THP-1 macrophages. M0 THP-1 macrophages were treated with QPD or XBD at indicated concentrations for 24 h and then stimulated with or without 1 µg/ml LPS and 20 ng/ml IFN-γ for 1 h or 2 h. Total proteins were extracted to detect IκBα, phospho-IκBα, NF-κB p65, and phospho-NF-κB p65 by western blot **(A)**. Total RNA was extracted to detect NF-κB by RT-qPCR **(B)**. LPS+, treated with LPS and IFN-γ; LPS-, treated without LPS and IFN-γ. *** p<0.001 .

Activation of the transcription factor nuclear factor kappa B (NF-κB) is a key step in mediating the expression of various cytokines. To investigate whether the inhibition effect of QPD and XBD on cytokine production was related to the modulation of the NF-κB signaling pathway, we measured total IκBα, NF-κB p65, phospho-IκBα, and phospho-NF-κB p65 protein levels by western blot in THP-1 macrophages treated with LPS and IFN-γ. As shown in Figure 4A, the phosphorylation levels of IκBα and NF-κB p65 were elevated in the LPS-stimulated control groups, indicating successful activation of the NF-κB pathway following LPS stimulation. Both QPD and XBD treatments suppressed phosphorylation of IκBα and NF-κB p65 at 1 h or 2 h post LPS stimulation. The levels of total IκBα were not changed following QPD or XBD treatment. However, both QPD and XBD treatments slightly inhibited the total level of NF-κB mRNA expression in the LPS-free group, which was further confirmed by RT-qPCR (Figure 4B).

**FIGURE 5:**
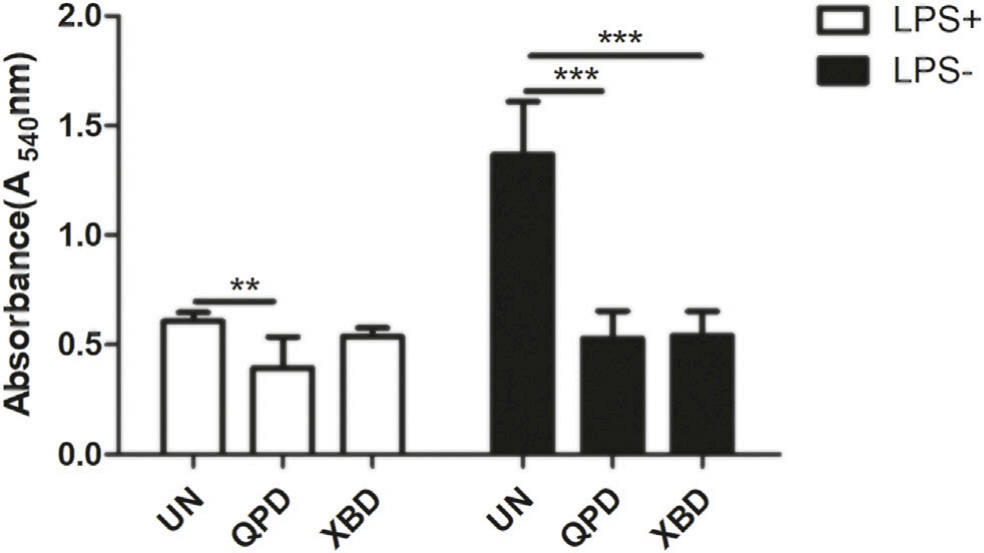
QPD and XBD inhibited pinocytosis activity of THP-1 macrophages. M0 THP-1 macrophages were treated with QPD or XBD (5%, v/v) for 24 h and then stimulated with or without 1 µg/ml LPS and 20 ng/ml IFN-γ for another 24 h before neutral red uptake assay. LPS+, treated with LPS and IFN-γ; LPS-, treated without LPS and IFN-γ. ** p<0.001 ; *** p<0.001 compared with UN or as indicated.

Soluble antigens have been proven to directly enter macrophages and thereby induce signaling activation to mediate macrophage polarization. We therefore tested whether QPD and XBD affect the pinocytosis activity of THP-1-derived macrophages using neutral red uptake assay. As shown in Figure 5, the pinocytosis activity was significantly inhibited by QPD or XBD treatment, especially in the LPS-free group. Moreover, the suppression effect of QPD or XBD on pinocytosis was less significant in the M1 polarized macrophages, which might be due to the blunted pinocytosis activity induced by LPS stimulation.

## Discussion

Cytokine storm is closely associated with the severity and mortality of patients with COVID-19 (Hu et al., 2021; Kim et al., 2021). Accordingly, anti-inflammatory therapies are of great importance in the management of patients with severe COVID-19. In our current study, we focused our investigation on macrophages because they play a key role in cytokine storms and are the major source of pro-inflammatory cytokines (Wang J. et al., 2020) including IL-6 and TNF-α. Although the detailed immune modulation mechanisms vary among viruses, the activation of multiple Toll-like receptors (TLRs) is involved in the induction of a cytokine storm. Recent studies revealed that SARS-CoV-2 induced inflammation *via* TLR2/4 activation (Bhattacharya et al., 2020; Choudhury et al., 2020; Khan et al., 2021; Zheng et al., 2021). Similarly, LPS stimulation could also activate TLR2/4 signaling in macrophages (Orecchioni et al., 2019; Feng TT. et al., 2020), which mimic the activation status induced by SARS-CoV-2 to some extent. Moreover, the expression profiles of the pro-inflammatory cytokines (e.g. IL-6, TNF-a, CXCL10, and so on) in THP-1 are very similar in both the LPS-stimulated group and the SARS-CoV-2 envelope protein-stimulated group (Chiok et al., 2021; Pantazi et al., 2021; Shirato et al., 2021). Based on this, we utilized LPS-stimulated THP-1 macrophages in the present study.

Our present data show that both QPD and XBD could directly suppress the production of IL-6 and TNF-α in THP-1-derived macrophages, indicating a TCM-induced inflammatory modulation effect. As a cytokine critical to mediate inflammation, IL-6 has pleiotropic activity (Tanaka et al., 2014) and may play an opposing role in the immune response to different viral infections (Gubernatorova et al., 2020). Clinical data (Feng X. et al., 2020; Chen et al., 2020; Wang et al., 2021a) have shown that COVID-19 patients, especially severe patients, experienced significantly elevated systemic levels of IL-6 compared to healthy controls. Therefore, IL-6 is considered to be a useful biomarker for predicting the severity of a SARS-Cov-2 infection, although the exact mechanism remains to be elucidated. In line with this, the therapeutic potential of IL-6 inhibitors (Gritti et al., 2020; Liu et al., 2020; Xu et al., 2020), such as Tocilizumab and Siltuximab, have been investigated clinically. At the same time, the effect of TCM on IL-6 has also been explored. Pharmacological assays *in vitro* demonstrated the effects of some TCM, such as Liu Shen capsules (Ma et al., 2020), ReDuNing injections (Ma et al., 2021), and a novel formula NRICM101 (Tsai et al., 2021), in suppressing the expression of IL-6, as well as TNF-α. QPD has also been shown to contribute to IL-6 production. Recently, Y Ren et al. (2020) have found that QPD inhibited the arachidonic acid (AA) metabolic pathway which was closely involved in IL-6 production. Ruocong Yang and colleagues (Yang et al., 2020) reported that one major compound in QPD, glycyrrhizic acid, could inhibit IL-6 production *via* Toll-like receptor signaling. In the current study, we present further evidence to support these earlier observations. It has been reported that LPS could induce the expression of IL-6 and TNF-α *via* the activation of the NF-κB signaling pathway (Koch et al., 2014; Lee et al., 2017). Accordingly, we tested the activation of the NF-κB signaling pathway in macrophages with or without QPD or XBD treatment, following LPS stimulation. We found that both QPD and XBD suppressed NF-κB signaling, to a striking degree. Moreover, network pharmacology studies (Li et al., 2021; Niu et al., 2021; Xia et al., 2021) have revealed that numerous active compounds in TCM have significant molecular binding affinities with IL-6 or could block IL-6 mediated JAK-STAT signaling pathway, raising another possibility for the anti-inflammatory activity of TCM.

Interestingly, our data also showed that QPD and XBD could inhibit the pinocytosis activity of THP-1-derived macrophages. It is already known that pinocytosis is involved in macrophage activation and polarization, and contributes to different immune responses. M Hashimoto et al. (2014) reported that soluble HIV-1 Nef protein entered M2 macrophages by macro-pinocytosis, driving them towards M1-like macrophages by activating the transforming growth factor (TGF)-β-activated kinase 1 (TAK1) cascade. Abraxane, a first-line drug for the treatment of pancreatic cancer, could exploit macro-pinocytosis for its entry into the macrophages to facilitate the differentiation into proinflammatory M1 phenotype (Cullis et al., 2017). Although more robust scientific evidence is needed, the possibility does exist that QPD and XBD may change macrophages’ response to the microenvironment *via* regulation of pinocytosis, leading to a more favorable prognosis in COVID-19 patients.

The diverse impacts of QPD and XBD on SARS-CoV-2 are consistent with the complicated constituents and compounds in the decoctions. In addition to immune regulation, TCM may also affect SARS-CoV-2 infection in other ways. 1) QPD exerts anti-viral effects *via* acting on several ribosomal proteins, resulting in suppressed viral replication (Alshaeri et al., 2020). 2) Lianhua Qingwen capsules could directly inhibit viral replication and lead to abnormal virus morphology (Runfeng et al., 2020). In addition, SARS-CoV-2 hijacked ACE2 to enter host cells (Zamorano Cuervo et al., 2020), which is very important for virus replication. The SARS-CoV-2 spike (S) protein is composed of two functional units: S1 which directly binds to ACE2, and S2 which is responsible for the fusion of virus and cellular membranes after being cleaved by TMPRSS2 (Zhang et al., 2020). Therefore, the ACE2/TMPRSS2 pathway is a promising target to block the early stages of SARS-CoV-2 infections (Monteil et al., 2020; Ragia et al., 2020). Our present data show that there was no significant difference in ACE2 and TMPRSS2 mRNA expression between the TCM-treated group and the control group. However, we failed to evaluate ACE2 or TMPRSS2 at the protein level, and the cellular location and enzymatic activity should be also considered. More evidence will be needed to reveal whether other phases of SARS-CoV-2 replication can be blocked by QPD and XBD.

TCM has been developed “from the clinic to the laboratory”, which is opposite to the “laboratory to the clinic” process in Western medicine. There is still a long way to go to understand the mechanisms underlying TCM, including the action of QPD and XBD in COVID-19 therapy. *In vivo* animal studies are needed to complement the *in vitro* cell-based experiments, and the roles of the individual components of the decoctions should be evaluated, while the synergistic effects of different compounds also need to be explored in the future.

In conclusion, we demonstrated a significantly decreased IL-6 and TNF-α production in response to LPS stimulation in QPD- and XBD-treated macrophages, where NF-κB signaling may be the key regulator in the present study. Moreover, both QPD and XBD inhibited the pinocytosis function of THP-1-derived macrophages. Because macrophages are one of the most important effectors involved in the process of cytokine storms, we speculate that the QPD and XBD can inhibit the inflammatory phenotype of macrophages, reducing the risk of a deleterious, hyper-activated inflammatory response. Our current results partly explain the efficacy of QPD and XBD in the treatment of COVID-19 patients, especially in severe patients.

## Data Availability

The original contributions presented in the study are included in the article/Supplementary Material, further inquiries can be directed to the corresponding authors.
